# Microbial Leakage through Three Different Implant–Abutment Interfaces on Morse Taper Implants In Vitro

**DOI:** 10.3390/dj12070226

**Published:** 2024-07-19

**Authors:** Ricardo Faria Ribeiro, Victor Barboza da Mata, Lucas de Oliveira Tomaselli, Anselmo Agostinho Simionato, Emerson de Souza Santos, Adriana Cláudia Lapria Faria, Renata Cristina Silveira Rodrigues, Cássio do Nascimento

**Affiliations:** 1 Department of Dental Materials and Prostheses, School of Dentistry of Ribeirao Preto, University of São Paulo, Ribeirão Preto 14040-904, Brazil; vctmata@hotmail.com (V.B.d.M.); tomaselli@usp.br (L.d.O.T.); anselmo.simionato@usp.br (A.A.S.); adriclalf@forp.usp.br (A.C.L.F.); renata@forp.usp.br (R.C.S.R.); 2 Department of Clinical Analysis, Toxicology, and Food Science, School of Pharmaceutical Sciences of Ribeirao Preto, University of Sao Paulo, Ribeirão Preto 14040-903, Brazil; emerson.souza.santos@usp.br

**Keywords:** dental implants, dental implant–abutment design, microbiological phenomena

## Abstract

The objective of this study was to evaluate microbial leakage by means of genome counts, through the implant–abutment interface in dental implants with different Morse taper abutments. Fifty-six samples were prepared and divided in four groups: CMC TB (14 Cylindrical Implants–14 TiBase Abutments), CMX TB (14 Conical Implants–14 TiBase Abutments), CMX PU (14 Conical Implants–14 Universal Abutment) and CMX U (14 Tapered Implants–14 UCLA Abutments). Assemblies had their interface submerged in saliva as the contaminant. Samples were subjected either to thermomechanical cycling (2 × 10^6^ mechanical cycles with frequency of 5 Hz and load of 120 N simultaneously with thermal cycles of 5–55 °C) or thermal cycling (5–55 °C). After cycling, the contents from the inner parts of assemblies were collected and analyzed using the Checkerboard DNA–DNA hybridization technique. Significant differences in the total genome counts were found after both thermomechanical or thermal cycling: CMX U > CMX PU > CMX TB > CMC TB. There were also significant differences in individual bacterial counts in each of the groups (*p* < 0.05). Irrespective of mechanical cycling, the type of abutment seems to influence not only the total microbial leakage through the interface, but also seems to significantly reflect differences considering individual target species.

## 1. Introduction

With the continuous advancement of rehabilitation materials and techniques, implant-supported restorations have been extensively used in dentistry with a very high predictability rate of both success and survival [1]. Although the use of osseointegrable implants can be considered safe and predictable, the oral environment presents itself as one of the most challenging places in the human body, bringing mechanical, thermal and acidity variations and, mainly, microbial challenges. The use of two-part dental implants presents as advantages the reliable standard protocol and easy handling of peri-implant tissues, providing several possibilities of the transmucosal area, prosthetic heights, diameters and angulation. However, these systems still have a major concern related to the implant–abutment interface; to date, it has not been possible to make it impervious to the passage of microorganisms, including those that may cause peri-implant diseases [2].

The presence of microorganisms at this interface, if not controlled, can result in inflammatory processes and cause damage to peri-implant tissues as the bacterial presence activates inflammatory cells, inducing osteoclastic action and consequent bone loss [3]. Scanning electron microscopy images demonstrate the presence of bacteria at the implant–abutment interface in failed implants, suggesting that these bacteria might be part of the factors causing inflammation and consequent bone loss in the region [4].

It already seems clear from the literature that Morse taper connections tend to present a lower amount of bacterial penetration through the interface region, resulting in the reduced contamination of the inner parts of the implant components. Most of these studies have compared conical and external hexagon connections, but also often include an internal hexagon [2]. The ostensible comparison between these three renowned interfaces and the considerable number of works demonstrating the superiority of conical connections in the reduction of bacterial infiltrate can lead the reader, clinician or even researcher to the erroneous understanding that all conical connections behave in the same way. In a study, Bella et al. compared indexed (two-piece) and non-indexed (solid one-piece) conical connection abutments and found a statistical difference in the bacterial infiltrate between them [5]. Among conical connections, we can find numerous differences; the angulation of the cone in relation to the long axis of the implant, the fixation of the implant using a through screw (two-piece) or a solid abutment and the material that the abutment is made of. The macrogeometry of implants can also play a relevant role in the passage of microorganisms and other substances into the implants, since the distribution of forces throughout the systems can alter the micro-movements of the components and influence the microgaps into the interface.

Therefore, it is important to have knowledge about how the connections of three different abutments that are widely used in dental clinics behave: Universal abutment, widely used due to its simplicity, reliability and good adaptation; UCLA-type abutments, which, due to their low cost and because they represent a direct connection from the implant platform, are widely used in cases of reduced transmucosal tissue, but may represent a potential risk for the passage of microorganisms due to the finishing process and polishing, as seen in a study by Rismanchian et al. [6]; and the TiBase abutments that are gaining strength nowadays due to their versatility and the possibility of inserting oral rehabilitation through implants in the digital dentistry world, but their use on a large scale must be carried out with caution, since the literature shows differences in the misadaptation gap between different manufacturers of TiBase abutments [7]. Also, how the shape of the implants can influence the adaptation of the components and the passage of microorganisms after loading should be investigated.

The specificities of each system or components, such as changes in the insertion torque, through screw and composition of the implant alloys can generate different types of bacterial infiltrates and these can be associated with the inflammatory processes that give rise to peri-implantitis. Socransky et al. demonstrated that different bacteria are related to different clinical parameters, which allows us to relate certain bacterial complexes to different clinical situations [8]. The Checkerboard DNA–DNA hybridization detection methodology used in this study allows the simultaneous identification and quantitation of up to 45 different species of microorganisms in the same sample [9].

Knowledge of the behavior of different implants and components in the face of the passage of microorganisms can provide relevant information not only to encourage new research in this area of knowledge, but also help the rehabilitation dentist when making decisions in the phase of choosing implants and prosthetic components aiming for the greater predictability of long-term treatment success. In this context, the objective of this study was to evaluate the bacterial infiltrate recovered from three different conical connections (universal abutment, Co-Cr UCLA and TiBase 4CAD) after thermomechanical cycling. The hypotheses tested were that there would be differences in the bacterial quantitation between conical and cylindrical implants with different connections and both thermal and thermomechanical simulations would influence the results.

## 2. Materials and Methods

### 2.1. Experimental Design

This is a randomized in vitro experimental study, with parallel groups, investigating the leakage of microorganisms through the implant–abutment interface of a system with cylindrical and conical implants, with Morse taper connection and different prosthetic abutments. The research was carried out at the Molecular Dental Diagnostic Laboratory of the School of Dentistry of Ribeirão Preto, University of São Paulo. Human saliva was used as contaminant media, the study protocol was approved by the Local Ethics Committee under the number CAAE: 25836819.2.0000.5419 and all experiments were carried out with the participants’ written consent.

### 2.2. Implant and Abutment Selection and Constitution of Study Groups

**Figure 1:**
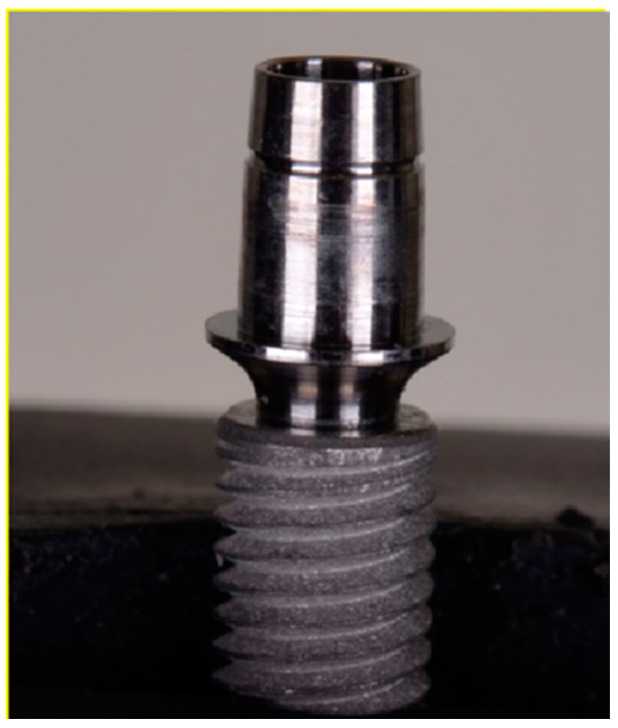
Tibase 4 CAD abutment.

**Figure 2:**
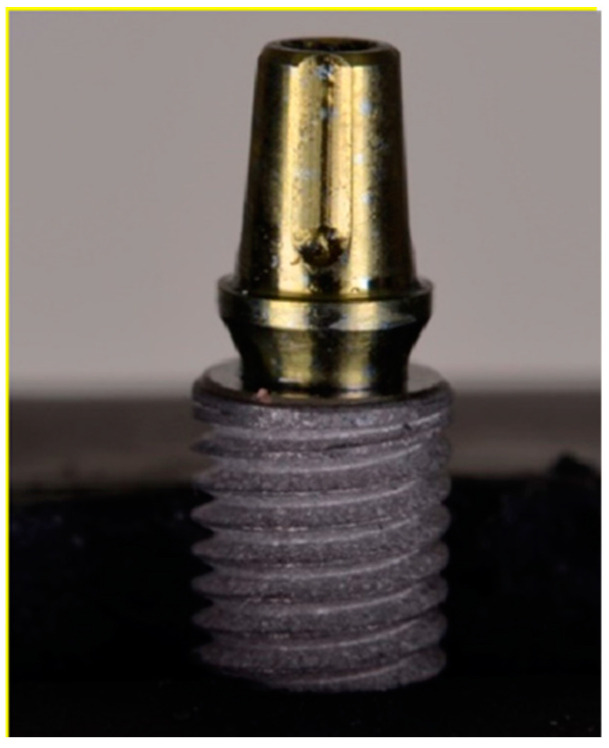
Universal Abutment.

**Figure 3:**
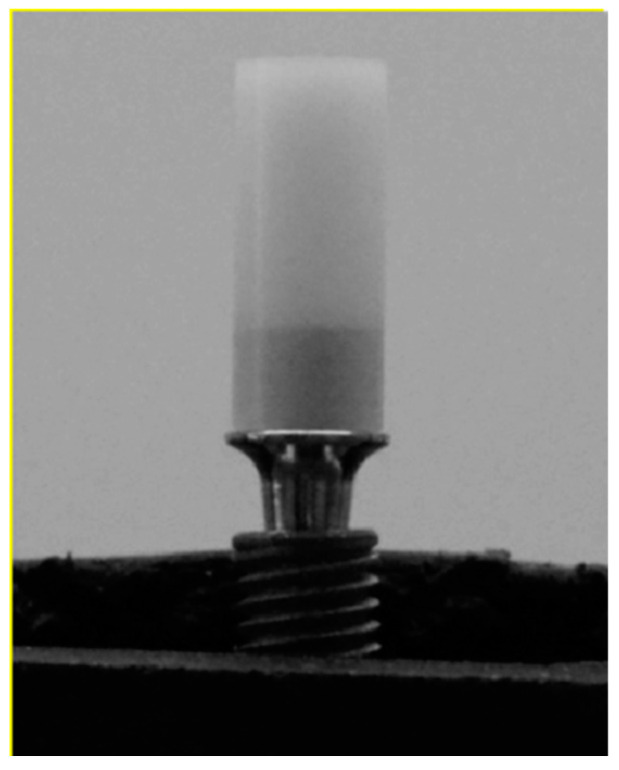
UCLA abutment.

This research involved the use of grade 4 titanium dental implants (*n* = 56) with Morse taper connection (Singular Implants, Natal, RN, Brazil), of which 42 had a conical shape (CMX; 4.0 × 13—Ref: 100.194) and 14 cylindrical (CMC; 4.0 × 13—Ref: 100.124). All implants used in the study are indicated for intraosseous use, with an internal cone angulation measuring 11.5°. The abutments investigated were as follows: (TB) Tibase 4 CAD CM N—1.5 mm (*n* = 28; Ref: 126.125; Figure 1), (PU) Universal abutment CM—3.3 × 4–1.5 mm (*n* = 14; Ref: 119.014; Figure 2) and (U) UCLA Co-Cr CM—1.5 mm (*n* = 14; Ref: 103.120; Figure 3). Among the 14 samples in each group, we separated 10 samples for the thermomechanical cycle and 4 samples we removed from the mechanical challenge, which then underwent solely thermal cycling.

### 2.3. Collecting Negative Control and Abutment Fixation

**Figure 4:**
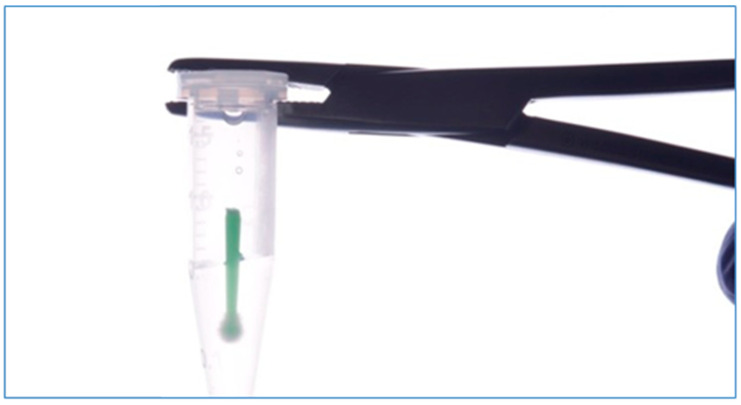
Sample taken from the inner part of the implants and components before thermocycling.

All the implants and components used were previously sterilized by gamma ray; however, to ensure that there were no pre-existing bacteria, before implant–abutment assembly, samples from the inner parts of the implants and the surfaces of the screws from all samples were collected with the aid of sterilized microbrushes serving as negative control for contamination, with a total of 56 samples obtained. Samples were stored individually in microtubes with 80 μL of TE buffer solution (10 Mm Tris-HCl, 1 Mm EDTA pH 7.6; Figure 4).

The implant–abutment set was connected using a digital torquemeter (Torque Meter TQ 8800, Instrutherm, São Paulo, Brazil) which was fitted to a delineator created in the Department of Dental Materials and Prosthetics of the School of Dentistry of Ribeirão Preto-USP to maintain standardized positioning of the prosthetic key for applying force (torque). The abutments were connected to the implants applying the torque load Ncm recommended by the manufacturer and experimental groups were as follows: CMC TB (32 Ncm), CMX TB (32 Ncm), CMX PU (32 Ncm) and CMX U (20 Ncm).

As recommended in the literature, after 10 min of applying the load recommended by the manufacturer, the retaining screw was retorqued applying the same load, aiming to avoid loss of torque resulting from the deformation and flow of the components [10,11].

All component attachment steps took place using a laminar flow, with the work surface disinfected with 70% alcohol and previously sterilized for 30 min by ultraviolet light. The glassware, tweezers and keys for applying force were previously sterilized in an autoclave and the researchers were equipped with personal protective equipment.

### 2.4. Polyurethane Bases and Sample Positioning

The 56 implant–abutment sets were embedded in polyurethane to be subjected to thermocycling and cyclic loading prior to the microbial contamination test. A silicone base was used as a mold to construct the test specimens. On a precision scale, 7.5 g of Polyol + 7.5 g of Isocyanate (Isocyanate F 160, Sika Axson, Madison Heights, MI, USA) were weighed, manipulated for 30 s, poured onto the silicone base and awaited the polymerization process, as recommended by the manufacturer.

To standardize the implants positioning on polyurethane base, an adjustable base parallelometer was used. The silicone base was positioned on the parallelometer base and the implant–prosthetic abutment set was connected to the mobile rod, ensuring the same position in all sets.

The positioning of the implants was carried out so that the prosthetic platform was at the level of the polyurethane surface, simulating an implant at bone level.

### 2.5. The 3D Crowns

**Figure 5:**
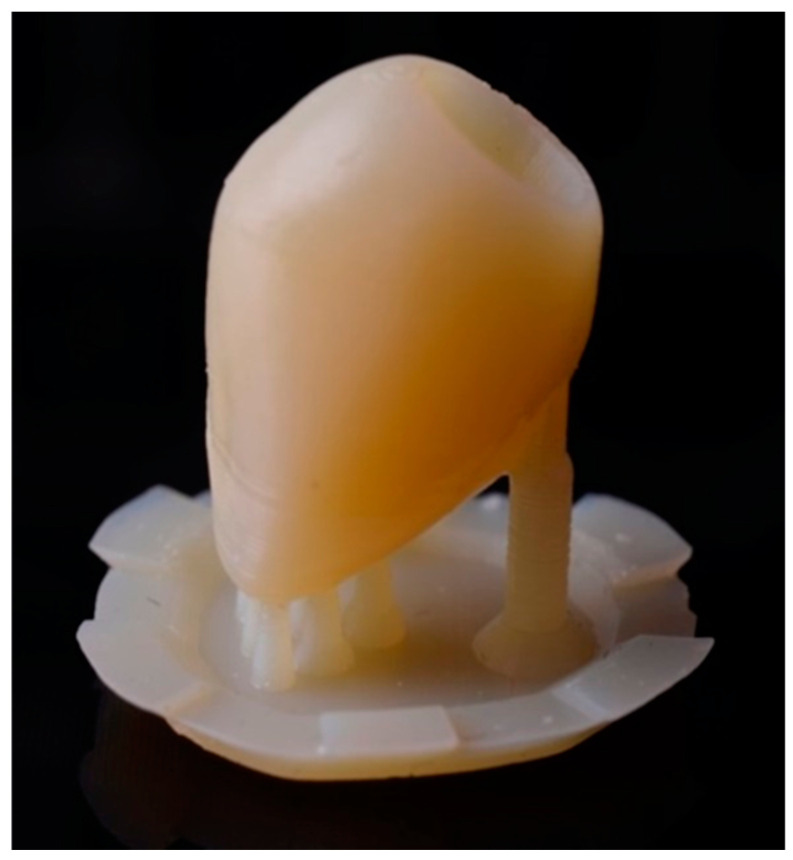
Three-dimensional resin-printed crowns in the shape of a maxillary canine.

Three-dimensional resin crowns in the shape of a maxillary canine were fabricated using the Autodesk MeshMixer version 3.5 software (Autodesk Inc., San Rafael, CA, USA) for the cyclic load simulation on the assemblies. A profile projector (Profile Projector Model 6C, Nikon, Tokyo, Japan) was used to standardize the crown dimensions for the different abutments. The crowns (Figure 5) were printed on the Phrozen Sonic 4K 3D printer [Phrozen Tech. Co., Hsinchu City, Taiwan (R.O.C.)] with 3D temporary resin (Printax, Phrozen Tech. Co.). The loading applicators, 15 mm in diameter × 15 mm in width and with an angulation in the internal region of 10° that were coupled to the fatigue machine, were also manufactured in printed 3D resin, following the same protocol as the crowns.

### 2.6. Saliva Collecting

To collect the unstimulated human saliva used as a contaminant media for the implant assemblies, 20 healthy adults were selected among students and employees of the university itself. Participants of both genders with no signs and symptoms of systemic diseases or infections in the oral cavity were included. Furthermore, a group of participants with similar ages and environmental conditions was sought, aiming for uniform saliva samples. Collections were always carried out at the same time and place.

**Figure 6:**
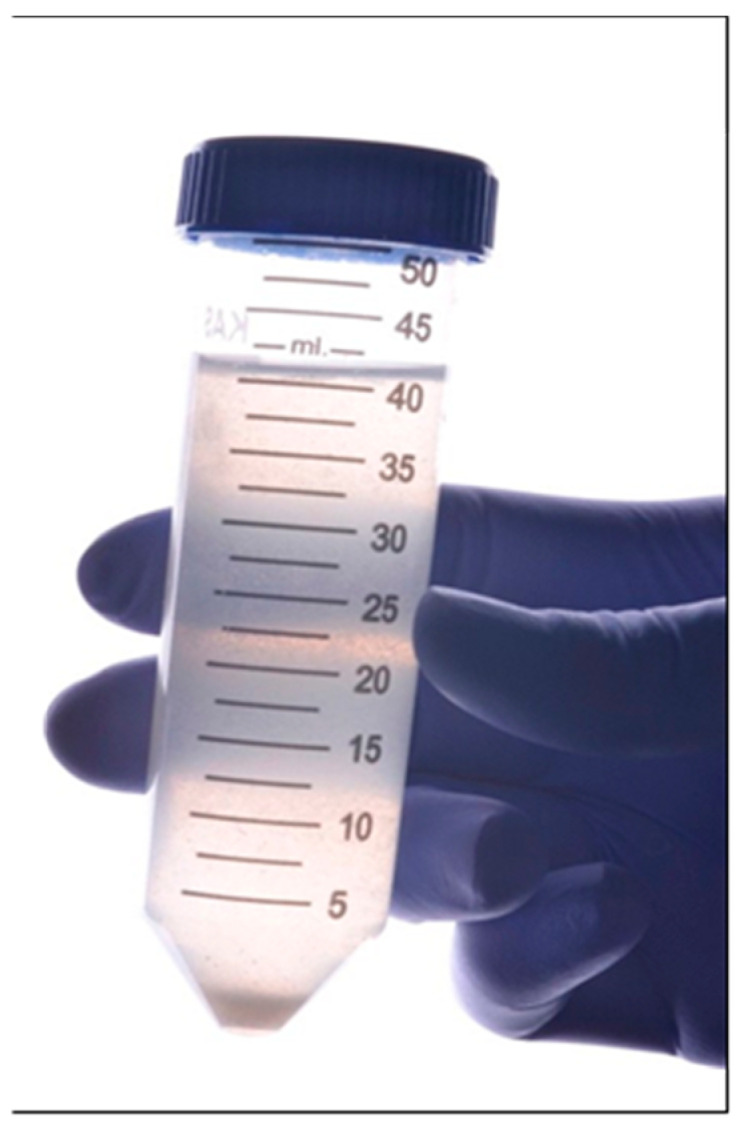
Tube with saliva collected from participants that served as a contaminant for the samples.

Each participant contributed 6 mL of saliva. After individual collecting, samples from the 20 participants were pooled and homogenized for 3 min and transferred to a single tube constituting the contaminant media and stored in a bacteriological oven at 37 °C throughout the cyclic loading and thermocycling test (Figure 6).

### 2.7. Final Preparation of Specimens and Contamination Test

After re-torque, the screw access channel was sealed with Teflon tape and light-polymerizable microhybrid composite resin (Applic Flow-Maquira), then the crown was cemented onto the components with Polyether (Impregum soft—3M).

**Figure 7:**
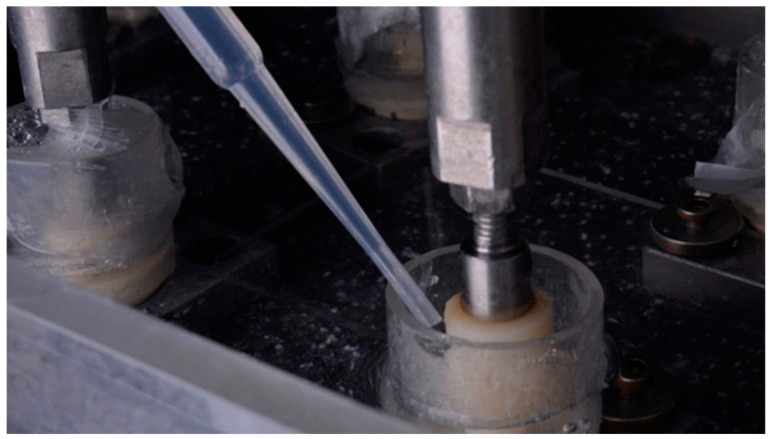
Pipette inserting the collected saliva into the implant–abutment interface before the thermomechanical test.

Rubber tubes were fixed to the polyurethane with water-based silicone and, in this way, the implant–abutment assembly was isolated from the external environment in a reservoir for placing saliva during the cyclic load application test. Next, 3 mL of saliva was inserted into each of the reservoirs (Figure 7). The volume of saliva added was sufficient to cover the implant–prosthetic abutment connection interface without reaching the access hole of the abutment fixation screw, in order to minimize the passage of microorganisms from saliva through any route other than the implant–abutment interface. At the end of the cyclic loading test, samples (*n* = 10) from the tube containing the human saliva used as contaminant media were collected and used as positive control to verify the microbial profile of the saliva before and after the loading test period.

### 2.8. Thermomechanical Cycling

Ten implant–abutment sets from each group were subjected to the cyclic load test. The chewing mechanical fatigue machine (BIOPDI—Equipment for research into medical and dental materials, São Carlos, Brazil) was used to simulate human chewing, which is in the Biomechanics Laboratory of the School of Dentistry of Ribeirão Preto. This machine made it possible to conduct dynamic tests on 10 specimens simultaneously. Force applicators were made on the 10 pistons using a 3D resin printer. The 10 pistons used to apply load acted independently on each specimen.

The loading of each specimen during the simulation of chewing cycles occurred through a system of springs. The load was applied during thermal cycles of 5–55 °C (25 s of filling, 5 min of residence and 35 s of emptying). This loading application process was carried out automatically.

**Figure 8:**
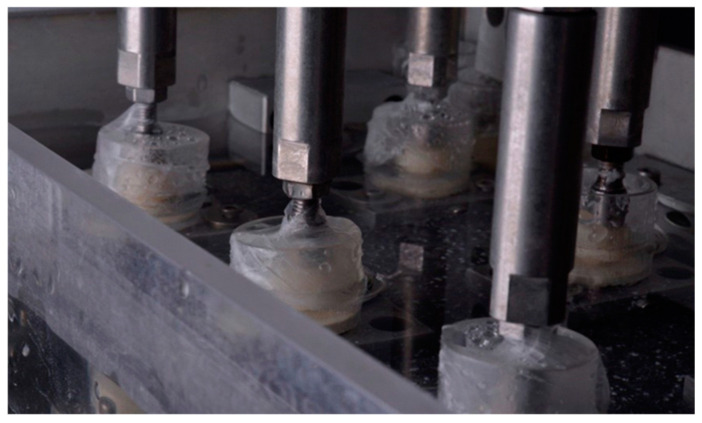
Thermomechanical cycling test machine with the specimens fixed and the load applicators.

The test specimens’ sets, as seen in Figure 8, immersed in saliva were fixed to the base of the testing machine, remaining juxtaposed, since their polyurethane bases were built with the same dimensions as the machine’s fixing niches. The specimens were positioned and the load was applied incisally from the prosthetic crown to the long axis of the implant. The entire system was sealed with flexible adhesive film (Parafilm, Neehan, WI, USA), which allowed its isolation from the external environment.

The machine was programmed for the controlled application of a load of 120 N through the loading applicators in the incisal region of each crown. The 2 × 10^6^ cycles were performed, with a frequency of 5 Hz. Simultaneously, the base to which each specimen was fixed performed horizontal movements of 1 mm to the right side and 1 mm to the left side, replicating the excursive mandible movements.

### 2.9. Thermocycling Test

The specimens were also tested for the passage of microorganisms when subjected to thermal cycling only, without cyclic loading. Four implant–prosthetic abutment sets from each of the 4 groups analyzed were fixed to polyurethane and submerged in 3 mL of human saliva and coupled to the base of the fatigue machine, undergoing only thermocycling, without applying force.

### 2.10. Assessment of Microbial Leakage Using Checkerboard DNA–DNA Hybridization Method

Samples from the inner part of the implant–abutment assemblies subjected to either thermocycling tests with cyclic load or thermocycling were collected to identify and quantify the microorganisms that penetrate through the interface. For this, the Checkerboard DNA–DNA hybridization method was used according to Socransky et al. with a modification by do Nascimento et al. [9,12].

Before collecting, the rubber tubes were removed from the polyurethane base of assemblies and all the external surfaces of experimental and control sets were carefully washed with 70% alcohol and dried with sterilized gauze pads. The crowns were then removed from the prosthetic abutment with the aid of hemostatic forceps. The sets were reopened to collect content from the inside of the implants and fixation screw threads using sterile microbrushes. The samples were individually inserted in microtubes containing 150 μL of TE buffer solution and stored at 4 °C until laboratorial processing.

For this study, 40 different microbial species were selected, ranging from the initial colonizers of the microbial biofilm to species considered pathogenic for the development of periodontal and peri-implant diseases. Four species of Candida, commonly detected in the oral cavity, were included: *Candida tropicalis*, *Candida glabrata*, *Candida dubliniensis*, *Candida albicans*, *Streptococcus pneumoniae*, *Streptococcus gallolyticus*, *Veillonella parvula*, *Treponema denticola*, *Tannerella forsythia*, *Streptococcus sobrinus*, *Streptococcus sanguinis*, *Streptococcus salivarius*, *Staphylococcus pasteuri*, *Streptococcus parasanguinis*, *Streptococcus oralis*, *Streptococcus mutans*, *Solobacterium moorei*, *Streptococcus mitis*, *Streptococcus constellatus*, *Staphylococcus aureus*, *Pseudomonas putida*, *Prevotella nigrescens*, *Parvimona micra*, *Prevotella melaninogenica*, *Prevotella inter media*, *Porphyromonas gingivalis*, *Porphyromonas endodontalis*, *Peptostreptococcus anaerobius*, *Pseudomonas aeruginosa*, *Mycoplasma salivarium*, *Lactobacillus casei*, *Klebsiella pneumoniae*, *Fusobacterium nucleatum*, *Enterococcus faecalis*, *Eikenella corrodens*, *Escherichia coli*, *Campylobacter rectus*, *Capnocytophaga gingivalis*, *Bacteroides fragilis* and *Aggregatibacter actinomycetemcomitans*.

### 2.11. Data Analysis

Data from Checkerboard DNA–DNA hybridization were analyzed using the CLIQS 1D software (Totallab, Newcastle, UK). The approximate number of microbial cells (genome counts) present in each investigated sample was estimated by comparing the intensity of the hybridization signals obtained by the intersection of the samples against the labeled probes in relation to the intensity of the standards containing 10^5^ and 10^6^ cells from each of the 40 target species.

Descriptive analysis of the data was performed, including point estimators such as means, medians and quartiles (first quartile—Q1—and third quartile—Q3), according to their distribution and variance of experimental errors. Data normality was verified by visual inspection of density graphs, histograms and Q-Q plots and confirmed by the Shapiro–Wilk significance test. Homoscedasticity was verified by Levene’s test. The analysis of the effect of the abutments and thermocycling or cyclic load tests on the count of microorganisms was carried out using the non-parametric and multifactorial Brunner–Langer method with Bonferroni adjustment. Considering the multifactorial and correlational nature of microbiological data, the Generalized Estimating Equations—GEE—model was used to compare the microbial profile between groups. Data were processed using the statistical software R (R software, version 4.1.0; R Foundation for Statistical Computing, Vienna, Austria) and differences were considered significant at *p* value < 0.05.

## 3. Results

None of the negative control samples from all of the evaluated groups, collected from inside the implants prior to the thermocycling and cyclic loading test, showed positive results for microbial presence, ensuring the sterilization effectiveness carried out by the manufacturer.

After the thermocycling and cyclic loading test, all implant–abutment sets of all abutments investigated showed the presence of microorganisms, totaling 2,041,551 recorded genomes. A similar result was observed for the abutments that were subjected only to the thermal test, with a total of 1,997,290 genomes. Among the groups subjected to thermocycling and cyclic loading, the median total genome count, in order from highest to lowest, was CMC TB (627,805), CMX TB (572,820), CMX PU (519,282) and CMX U (321,644). Among the groups subjected only to thermal cycling, the medians were CMC TB (617,112), CMX TB (550,809), CMX PU (513,847) and CMX U (315,522).

**Figure 9:**
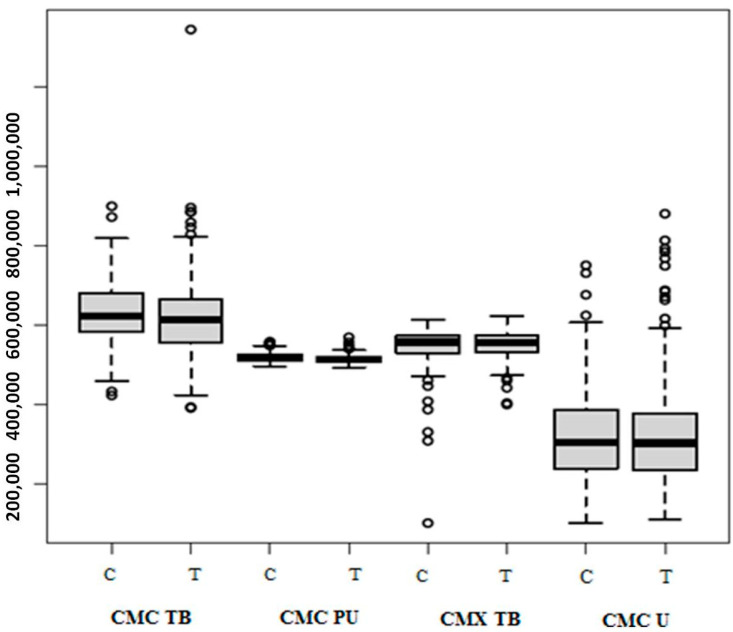
Box Plot with median, maximum and minimum values and interquartile range of quantification of total genomes of the 40 target species identified in the screw threads and inside the implant of the groups subjected to cyclic loading (C) and thermocycling (T).

The groups investigated showed significant differences in the genome counts (WTS and ATS; *p* < 0.005). Figure 9 illustrates the median, maximum and minimum values and interquartile range of the total genome counts of microbial cells from samples collected inside the implants and fixation screws subjected or not to cyclic loading, while in Table 1, Table 2, Table 3 and Table 4, the values referring to the individual count of each of the 40 target species evaluated in the study are described. The lowest values recorded between the thermocycling and cyclic load groups were the CMX U group, while the CMC TB and CMX TB groups presented the highest values (Tukey; *p* < 0.005). Among the thermocycling groups, the one that presented the best results was also the CMX U group.

The Generalized Estimation Equations (GEE) method showed that there were significant differences in the counts of the different target species of the study between the groups after the cyclic load test (*p* < 0.05). As shown in Table 1, the microorganisms with the highest counts found in the CMC TB group subjected to thermal cycling and cyclic loading were *S. sobrinus* (750,250), *T. forsythia* (746,543), *T. denticola* (660,446), *P. melaninogenica* (658,453), *S. moorei* (655,223) and *S. sanguinis* (651,256). Among the groups subjected to thermal cycling, the highest counts were for *S. sobrinus* (820,197), *T. forsythia* (723,360), *E. faecalis* (675,718), *C. gingalis* (667,024), *E. corrodens* (662,937) and *melaninogeneca* (659,362).

As displayed in Table 2, the microorganisms with the highest counts found in the CMX TB group subjected to thermal cycling and cyclic loading were *K. pneumoniae* (581,477), *L. casei* (580,824), *E. corrodens* (578,317), *C. rectus* (577,864), *F. nucleatum* (575,682) and *E. faecalis* (573,064). Among the groups subjected to thermal cycling, the highest counts were for *S. oralis* (580,457), *S. sobrinus* (580,099), *E. corrodens* (578,561), *K. pneumoniae* (578,279), *L. casei* (576,619) and *B. fragilis* (575,094).

As presented in Table 3, the microorganisms with the highest counts found in the CMX PU group subjected to thermal cycling and cyclic loading were *C. tropicalis* (535,348), *C. Glabrata* (524,599), *T. forsythia* (524,148), *P. melanogenica* (520,954), *S. sobrinus* (520,340) and *C. albicans* (519,744). Among the groups subjected to thermal cycling, the highest counts were for *C. tropicalis* (546,923), *C. Glabrata* (528,696), *S. sobrinus* (526,706), *P. putida* (525,870), *T. forsythia* (525,246) and *C. dubliniensis* (525,243).

As shown in Table 4, the microorganisms with the highest counts found in the CMX U group subjected to thermal cycling and cyclic loading were *P. melaninogenica* (591,295), *C. rectus* (477,979), *C. gingivalis* (425,489), *B. fragilis* (408,566), *K. pneumoniae* (402,078) and *S. oralis* (390,849). Among the groups subjected to thermal cycling, the microorganisms with the highest counts were *P. melaninogenica* (632,014), *B. fragilis* (470,874), *P. nigrescens* (442,321), *Candida dubliniensis* (434,964), *Candida albicans* (378,853) and *P. putida* (366,827)

**Figure 10:**
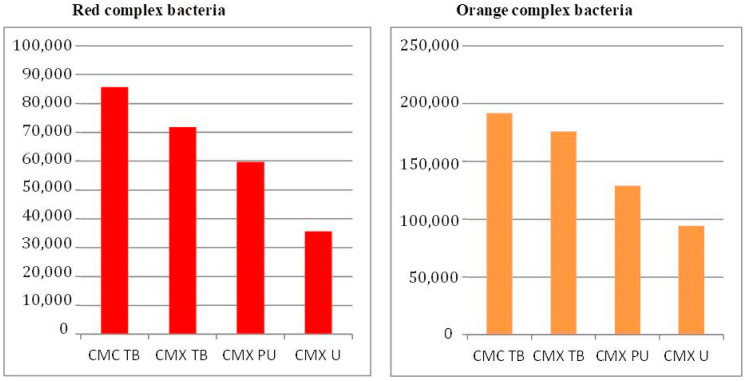
Bacterial count (total genomes) after the thermomechanical loading assay.

**Figure 11:**
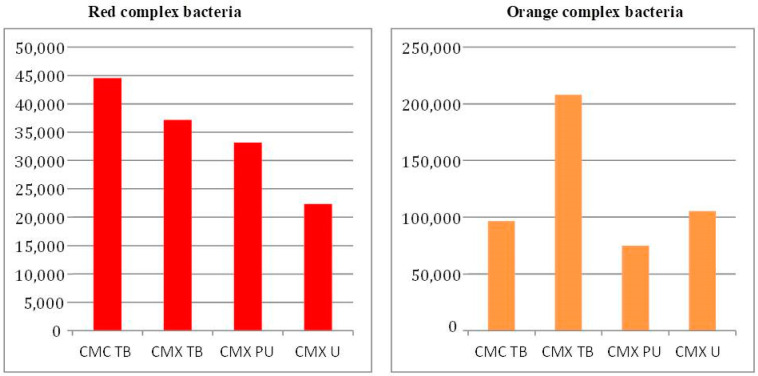
Bacterial count (total genomes) after the thermal cycling assay.

Figure 10 and Figure 11 illustrate the distribution of bacterial species according to the Socransky red and orange complexes, respectively, in the investigated groups subjected or not to cyclic loading [13].

The results indicate that the amount of red complex bacterial genomes detected in the groups after the cyclic loading test was much higher than the amount observed in the groups subjected only to the thermocycling test. However, the distribution pattern of genomes in the groups is similar, with the highest values being observed for the CMC TB group and the lowest for the CMX U groups.

For microorganisms belonging to the orange complex, the number of genomes observed in the two experimental situations, with or without charge, was similar. However, the distribution profile between the groups was different in both situations; for the groups subjected to the cyclic load test, the pattern was similar to that of the red complex, with the highest counts observed for the CMC TB group, followed by CMX TB, CMX PU and CMX U. For the groups subjected only to the cyclic load test, thermocycling, the pattern was very different, with the CMX TB group presenting a much higher count of total genomes when compared to the other groups that were similar.

## 4. Discussion

The present study evaluated the microbial leakage through the implant–abutment interface in conical connection implants with different prosthetic abutment designs after thermal cycling associated or not with cyclic loading. To achieve this, the assemblies were subjected to a cyclic load test simulating human chewing and the Checkerboard DNA–DNA hybridization technique was used to identify and quantify the presence of up to 40 microbial species that commonly colonize the oral cavity, including bacteria and fungi that are associated with the inflammatory processes that may cause periodontal and peri-implant diseases.

Dental implant systems are conventionally used in two parts: the implant and the prosthetic component (abutment) that adapts to the implant and receives prosthetic rehabilitation. Studies point to the conical connection as having the best mechanical/biological performance when compared to others existing connection designs. The junction between these two pieces, even if very well adapted, results in spaces that can favor the colonization and multiplication of microorganisms present in the oral cavity [14,15,16]. The hypothesis tested in this study was confirmed since the results demonstrate that all types of abutments and implants investigated have not avoided the passage of microorganisms through the interface, even when not subjected to the cyclic load test. Despite advances with dental implants and their connections, the occurrence of microbial infiltration through this interface is expected, since the size of the spaces reported in the literature can vary between 0.1 and 10 µm and the average diameter of the smallest bacteria present in the oral cavity varies between 0.2 and 1.5 μm in width and 2 and 10 μm in length [15,16,17,18]. Additionally, the micro-movements that occur between the components favor the opening of existing spaces [19,20]. Therefore, the null hypothesis established for this study was not confirmed, since there were significant differences in the microbial profile for the different groups investigated.

Although Morse cone connections are considered the most stable and have the least infiltration potential between implant components, the presence of microorganisms colonizing the interior of implants has been frequently reported in the literature, even in experimental tests with the absence of load application [3,14,21,22]. The implant–abutment interface in this connection design has friction adjustment and when this assembly is subjected to load, the spaces present can also be enlarged as a result of micro-movements, resulting in the infiltration of microorganisms and their fluids into the implant and vice versa [2,23,24,25]. Furthermore, the imprecise machining of the internal parts of the implant and the prosthetic abutment does not allow a sufficient contact area between the surfaces to provide an effective seal and may contribute to the occurrence of micro-leakage [26,27].

Teixeira et al. [28] found a percentage of *S. aureus* infiltration in 77% of implants with morse cone connections and 100% in internal hexagon-type connections. The results described in the literature showed that the Morse cone-type connection, when compared to the internal hexagon, presented a greater sealing capacity; however, it was not able to prevent the passage of bacteria and fluids through the connection interface [28,29,30].

Studies in the literature have already demonstrated the presence of more than 700 different species of microorganisms colonizing the tissues of the oral cavity, including viruses, protozoa, fungi and bacteria that cohabit in homeostasis [31]. When the biological balance is disrupted, inflammatory processes can occur with the consequent development of oral diseases [32]. Socransky et al. classified the various microorganisms into distinct microbial complexes, namely purple, green, orange, yellow and red, associated according to the bacterial virulence for periodontal disease. The purple, green and yellow complexes showed strong associations with each other and were less associated with the red and orange complexes, which present the bacterial species most closely related to disease conditions [13,33,34].

All target species proposed to be investigated in this study were found inside the implants of the different groups studied, both in situations involving the application of the cyclic load and in groups subjected only to thermocycling. The species *T. denticola*, *T. forshytia* and *P. gingivalis*, which are part of the red complex, and the species *S. constellatus*, *P. nigrescens*, *P. intermedia* and *C. rectus*, from the orange complex, are directly related to periodontal and peri-implant disease and were detected in moderate quantities inside the implants. Microorganisms from the red complex are almost always found in the presence of the orange complex, as they precede colonization by species from the red complex [13].

Comparing the count of red and orange complex microorganisms inside the implants, the thermal group with the cyclic load presented the highest values of bacterial genomes. However, the distribution pattern of genomes in the groups is similar, with the highest values being observed for the CMC TB group and the lowest for the CMX U groups. The cyclic loading condition may have caused micro-movements of the components during mechanical loading which facilitated the microbial passage at the implant–prosthetic abutment interface into the implant, as demonstrated in other studies [16,26].

Despite the lower microbial count, the condition of the group without the load simulation did not prevent infiltration at the implant–prosthetic component interface and, consequently, colonization inside the implant. Among the abutments of the static group, the CMC TB group also presented the highest count of genomes of bacteria from the red group (*T. denticola*, *T. forshytia* and *P. gingivalis*), while for microorganisms belonging to the orange complex (*S. constellatus*, *P. nigrescens*, *P. intermedia* and *C. rectus*), the CMX TB group presented a higher total genome count than the other groups, which in turn had a similar count. Other studies also detected the presence of microorganisms, even in conditions without a load simulation [14,22].

In general, the prevalence of microbial species between the different abutments was different; the CMC TB group had the most prevalent species: *S. sobrinus*, *T. forsythia*, *T. denticola*, *E. faecalis* and *C. gingivalis*. In the CMX TB group, the most common species found were *K. pneumoniae*, *L. casei*, *E. corrodens*, *S. oralis* and *S. sobrinus*. In the CMX PU group, the most prevalent were *C. tropicalis*, *C. Glabrata*, *T. forsythia*, *P. melanogenica* and *S. sobrinus*. In the CMX U group, the species *P. melaninogenica*, *C. rectus*, *C. gingivalis*, *B. fragilis* and *P. nigrescens* prevailed. The amount of microorganisms between the groups also showed significant differences in the contamination values (*p* < 0.005), in addition to the aforementioned factors that lead to screw loosening which may have contributed in different ways between the groups to the microbial passage into the interior of the implants. Other factors such as the microorganism size, microgap size, surface topography, atomic interactions and surface free energy of the pillars can also justify the different quantities and species detected between the groups [15,16,17,18,35,36,37,38,39,40]. Works using similar methodologies to the present study have also demonstrated the passage of microorganisms from the external environment to the interior of implants [9,14,21,41,42].

Overall, periodontopathogenic species belonging to the genera Porphyromonas, Tannerella and Treponema were found at moderate levels in the thermal cycling and cyclic loading groups. The presence of these pathogenic species is threatening when a microbial imbalance occurs or the host presents susceptibility, as proposed by the “ecological plaque theory”, which presents biofilm-mediated diseases as a result of an imbalance in the host’s microbiota [43,44,45].

In addition to bacteria, the presence of some fungi was also found inside the implants. The literature shows that the species *C. topicalis*, *C. albicans*, *C. glabata* and *C. dubliniensis*, identified in the groups studied, play a fundamental role, as opportunists in the constitution of biofilm in association with bacteria, playing a relevant role in the pathogenesis of peri-implantitis [46,47]. The most prevalent fungal species in the group subjected only to thermal cycling was *C. dubliniensis* and in the thermal group with a cyclic load, *C. glabata*. The species of fungi investigated in this study belong to the same genus, Candida, and, therefore, have many similarities in the constitution of their genetic material, mainly regarding their size and diameter. The differences in prevalence found for these species in the different groups in our study, as well as the differences observed for bacteria belonging to the same genus, need to be better investigated in future studies. One possibility could be the differences in the electrostatic potential and atomic interactions that occur between different microorganisms and substrates.

It is important to highlight that detecting microorganisms inside implants is not the confirmation of peri-implant disease, but rather a situation that can substantially increase the risk, since several other factors are necessary for the onset of the disease, with its etiology being multifactorial [48,49,50].

The method used to investigate the presence of micro-leakage in this study was Checkerboard DNA–DNA Hybridization, which has been widely used to detect and quantify species that harbor different sites in the oral cavity [51,52]. As it is a method based on identifying the genetic material of target species, it allows detecting viable or non-viable species within a biofilm. The detection of non-viable species is a very important factor, since just the presence of the bacterial cellular structure and its degradation products already pose a risk to peri-implant structures, as they serve as a substrate for other bacteria [41,53]. This method’s main characteristic is the speed and simultaneous identification of several species of microorganisms. Despite the excellent results provided, this detection method also has limitations such as reduced sensitivity, since the presence of microorganisms in concentrations lower than 10^4^ cells does not result in detectable or reproducible signals and only detects species that had the probes prepared from the DNA of the species defined as targets in the study [8]. Therefore, non-cultivable species or those that have not yet had their genome determined are not detected by this method. Furthermore, there is the possibility of the nonspecific binding of the probe labeling reagent with other macromolecules when the proportion of DNA is low [8]. Despite this, several studies have used this methodology to prove the micro-infiltration of microorganisms through the implant–prosthetic abutment interface [8,14,26,54].

The results of this study and many others, including literature reviews, demonstrate that despite presenting fewer occurrences of bacterial infiltration, conical connections are not capable of completely sealing the implant–abutment interface, making it vulnerable to the consequences of allowing a bacterial presence near this region. This finding leads us to question whether we should seek a different clinical approach, which seeks to learn how to deal with bacterial infiltration. In other words, we should seek a maintenance protocol that aims to control bacteria in the interface region. Sinjari et al., in 2018, published a study that provides important information about the implant–abutment interface. In the double-blind, controlled, randomized and prospective study Sinjari et al., with the objective of evaluating marginal bone loss, selected patients with prosthetic rehabilitation needs of a single element and separated the treatment times in five phases: t0—Implant placement surgery, t1—Reopening of the surgical room after 8 weeks, t2—Temporary placement after 12 weeks, t3—placement of final restoration after 14 weeks, t4—follow-up after 1 year. During the surgical procedure phases, however, one group (B) received cleaning of the interface region, carried out with gel 0.20% chlorhexidine, and the other group (A) received cleansing with placebo gel, without the inclusion of an antimicrobial agent. Bone loss analyses were performed during each phase of the study and the results demonstrate statistical differences in all surgical phases with greater bone loss for the group that received cleansing with the placebo gel. At t0, bone growth was observed in the test group and bone loss in the control group. In the subsequent periods, bone losses were observed for both groups, but always with statistical differences for lower loss in the test group [55,56,57,58,59].

Therefore, it can be understood that microbial infiltration through the implant–abutment interface is a problem still present in implant-supported rehabilitations, even when associated with implants with Morse cone connections, and this infiltration, if not minimized or controlled, can, in the long term, result in compromising the clinical success of the treatment if an imbalance of the associated oral microbiota occurs. More studies need to be conducted in order to clarify the relationship between the type of bacteria that develops and the clinical consequences, as well as studies that aim to develop interfaces that avoid, minimize or control the effects of the bacterial leakage.

## 5. Conclusions

Based on the results obtained in the present study, we can conclude that none of the implant–abutment combinations were able to prevent the microbial leakage through the interface. Mechanical cycling appears to play an important role in increasing the number of microbial counts. The design of the implant–abutment interface seems to be relevant to the type of microorganisms that penetrate and grow inside the implant.

## Figures and Tables

**Table 1:** CMC TB Group. Minimum (Min), 1st Quartile, median, mean, 3rd Quartile and maximum (Max) of the 40 target species detected in the screw threads and inside the implant.

Thermomechanical Loading							Thermocycling					
	Min	1st Quartile	Median	Mean	3rd Quartile	Max	Min	1st Quartile	Median	Mean	3rd Quartile	Max
*Candida tropicalis*	499,465	516,712	534,038	547,554	557,708	651,489	400,259	523,034	537,468	527,930	542,365	556,524
*Candida glabrata*	471,179	544,496	602,260	599,857	615,837	811,410	431,936	503,807	561,537	563,680	621,410	699,708
*Candida dubliniensis*	475,402	504,454	539,168	544,929	567,558	683,705	512,943	573,779	595,362	583,010	604,592	628,377
*Candida albicans*	503,754	514,799	534,297	552,731	584,323	649,825	480,652	512,195	523,533	551,795	563,133	679,463
*S. pneumoniae*	504,896	567,937	597,443	587,463	621,210	642,731	595,870	598,772	612,444	625,121	638,792	679,728
*S. gallolyticus*	507,870	546,434	579,044	587,641	615,521	697,752	589,889	638,313	655,077	644,569	661,366	678,234
*V. parvula*	539,243	590,354	597,497	606,167	635,940	658,358	567,120	585,344	593,449	588,217	596,322	598,849
*T. denticola*	483,362	578,609	656,805	660,446	763,000	828,167	561,773	586,472	647,304	653,646	714,479	758,203
*T. forsythia*	558,789	611,798	683,665	746,543	755,839	134,4715	659,659	704,875	722,092	723,360	740,576	789,596
*S. sobrinus*	661,538	699,976	726,574	750,250	806,908	895,655	691,838	786,421	844,923	820,197	878,699	899,104
*S. sanguinis*	530,248	594,529	638,470	651,256	652,013	819,170	504,070	633,840	691,386	651,957	709,502	720,986
*S. salivarius*	542,248	568,458	622,717	622,594	704,591	816,027	583,503	600,370	649,205	659,604	708,438	756,500
*S. pasteuri*	480,058	558,181	602,310	622,594	704,591	816,027	587,990	600,285	619,728	628,646	648,089	687,136
*S. parasanguinis*	536,446	5,707,749	629,047	630,151	667,066	757,201	550,919	605,175	636,416	640,084	671,325	736,586
*S. oralis*	474,413	568,393	652,822	634,822	704,656	762,701	530,628	543,113	591,199	604,967	653,053	706,844
*S. mutans*	487,655	546,186	620,196	603,589	658,292	703,153	457,503	467,810	509,795	538,970	580,955	678,787
*S. moorei*	567,414	624,352	644,285	655,223	660,830	787,577	566,710	577,428	638,138	642,531	703,241	727,137
*S. mitis*	479,870	570,950	608,519	613,243	623,248	858,306	574,245	607,696	630,267	632,519	655,090	699,222
*S. constellatus*	545,565	555,355	610,398	637,886	684,581	885,305	511,381	556,467	588,400	574,351	606,284	609,222
*S. aureus*	487,191	540,175	675,949	635,201	702,206	769,954	587,478	594,220	612,905	652,605	671,291	797,133
*P. putida*	516,300	619,464	650,428	647,966	698,450	786,884	558,804	586,594	633,324	624,951	671,681	674,352
*P. nigrescens*	501,925	574,848	610,700	608,865	637,100	707,094	587,177	632,085	655,643	640,732	664,291	664,466
*P. micra*	481,842	597,128	614,948	614,355	662,351	635,758	560,538	605,321	649,838	638,953	683,470	695,596
*P. melaninogenica*	581,484	642,451	656,114	658,453	679,316	748,853	525,768	573,214	651,422	659,362	737,570	808,836
*P. intermedia*	515,419	567,509	622,782	613,075	654,738	692,820	422,016	459,808	558,247	563,794	66,223	716,664
*P. gingivalis*	537,493	574,899	620,351	613,752	651,885	690,923	583,254	612,617	648,828	643,132	679,342	691,620
*P. endodontalis*	509,354	575,225	616,594	608,420	649,087	685,654	565,105	566,852	577,432	577,239	587,819	588,988
*P. anaerobius*	537,745	568,076	600,039	607,396	624,819	715,654	598,955	599,954	610,824	613,199	624,069	632,193
*P. aeruginosa*	481,647	582,853	640,709	624,453	681,893	751,444	565,730	591,237	599,990	606,724	615,477	661,188
*M. salivarium*	514,754	551,419	610,696	594,794	631,804	662,789	537,426	538,582	555,831	577,334	594,583	660,247
*K. pneumoniae*	478,531	542,816	599,017	589,771	634,740	681,970	511,323	573,528	616,948	618,669	662,088	729,458
*F. nucleatum*	390,358	509,930	539,019	552,677	624,528	662,581	629,171	632,180	655,032	657,829	680,680	692,081
*E. faecalis*	449,873	524,863	540,289	551,201	589,501	656,899	615,639	663,619	684,774	675,718	696,873	717,685
*E. corrodens*	392,714	578,014	610,590	580,026	627,132	684,806	567,392	586,274	666,448	662,937	743,111	751,458
*C. rectus*	423,191	526,447	663,110	615,102	676,448	752,398	612,597	633,473	640,603	647,565	654,695	696,458
*C. gengivalis*	474,206	591,170	650,446	641,002	674,923	787,770	608,877	628,253	668,521	667,024	707,292	722,178
*B. fragilis*	508,267	546,144	620,278	609,245	668,141	693,737	560,138	637,204	666,793	647,105	676,692	694,699
*Aaa*	476,387	569,010	612,387	615,976	632,509	521,162	521,162	571,564	633,465	622,224	685,226	698,605
*C. coli*	451,384	571,859	626,636	607,283	672,237	707,912	602,417	614,768	632,057	635,732	653,021	676,396
*L. casei*	565,001	581,528	646,157	638,162	683,994	724,165	479,441	588,346	646,438	624,235	682,327	724,623

**Table 2:** CMX TB Group. Minimum (Min), 1st Quartile, median, mean, 3rd Quartile and maximum (Max) of the 40 target species detected in the screw threads and inside the implant.

Thermomechanical Loading							Thermocycling					
	Min	1st Quartile	Median	Mean	3rd Quartile	Max	Min	1st Quartile	Median	Mean	3rd Quartile	Max
*Candida tropicalis*	496,592	515,731	526,960	525,193	53,851º	548,276	489,379	506,751	512,887	516,540	522,676	551,008
*Candida glabrata*	518,446	527,305	534,480	538,439	542,217	582,655	501,586	508,209	515,254	527,091	534,136	576,270
*Candida dubliniensis*	477,982	517,257	529,519	525,692	545,424	565,209	470,631	490,024	512,224	506,412	528,612	530,569
*Candida albicans*	473,474	494,370	523,611	516,886	529,699	572,128	492,392	499,021	510,583	517,413	528,993	556,093
*S. pneumoniae*	482,061	508,639	522,327	518,340	537,917	544,420	487,890	500,679	533,283	531,922	564,526	573,230
*S. gallolyticus*	399,066	507,491	528,371	520,918	554,330	565,274	489,946	498,905	521,177	525,008	547,280	567,733
*V. parvula*	512,181	526,347	536,916	531,959	540,894	542,268	386,188	452,881	487,084	477,951	512,154	551,449
*T. denticola*	543,743	558,818	563,354	568,994	572,934	613,872	542,853	560,909	569,516	565,391	573,999	579,678
*T. forsythia*	532,057	543,820	560,373	561,235	580,801	585,616	507,034	530,541	550,539	552,844	572,841	603,264
*S. sobrinus*	519,783	537,888	549,026	551,081	557,541	610,955	569,753	573,052	577,485	580,099	584,533	595,673
*S. sanguinis*	473,787	514,390	533,482	531,100	554,510	580,375	329,607	416,505	477,452	468,591	529,538	589,852
*S. salivarius*	475,515	525,172	534,335	530,916	544,926	559,998	515,742	518,324	531,689	533,916	547,281	556,545
*S. pasteuri*	459,655	494,762	521,916	516,966	540,400	560,938	481,242	485,587	491,090	489,862	495,364	496,024
*S. parasanguinis*	551,532	555,873	560,776	562,206	564,969	583,635	522,089	535,622	548,198	544,030	556,606	557,635
*S. oralis*	547,741	547,741	557,186	566,023	570,717	583,620	561,110	569,979	578,776	580,457	588,353	604,966
*S. mutans*	521,082	545,371	571,449	563,677	582,176	597,851	548,784	551,513	561,157	565,602	575,245	591,308
*S. moorei*	506,457	554,874	565,164	560,009	573,283	599,603	544,476	549,398	560,877	564,643	576,123	592,343
*S. mitis*	526,259	533,981	548,766	555,798	574,910	595,837	100,000	426,065	535,121	428,344	537,400	543,136
*S. constellatus*	527,892	560,203	569,269	566,945	574,598	592,919	547,831	562,346	567,613	563,918	569,184	572,616
*S. aureus*	548,861	556,266	563,362	564,513	574,890	578,580	526,178	569,788	584,634	571,746	586,592	591,541
*P. putida*	500,590	541,112	549,882	547,336	560,674	585,879	503,974	527,182	537,623	537,638	548,080	571,334
*P. nigrescens*	532,600	545,662	557,655	564,862	588,724	594,985	555,658	556,225	561,188	566,100	566,562	568,365
*P. micra*	402,709	550,502	561,360	543,071	568,187	622,768	307,382	493,785	557,719	501,157	565,090	581,808
*P. melaninogenica*	495,919	555,366	562,177	563,158	575,509	613,968	534,323	539,015	545,649	549,336	555,970	571,724
*P. intermedia*	544,669	557,460	569,073	571,801	580,986	613,662	564,974	569,463	571,896	572,860	575,293	582,673
*P. gingivalis*	489,725	504,471	535,813	527,119	546,902	554,464	507,347	518,645	536,165	538,127	555,646	572,833
*P. endodontalis*	441,086	486,754	502,020	496,015	507,733	529,601	407,109	447,178	468,629	476,382	497,832	561,159
*P. anaerobius*	479,681	521,592	532,805	539,360	537,340	573,804	529,878	549,986	559,938	553,762	563,714	565,295
*P. aeruginosa*	503,678	522,435	562,092	553,281	578,722	601,961	556,387	557,108	557,552	563,401	563,845	582,113
*M. salivarium*	521,811	535,519	565,304	558,432	574,149	591,321	541,602	559,289	572,156	567,911	580,778	585,729
*K. pneumoniae*	542,472	568,314	577,548	581,477	595,568	617,113	559,294	566,629	570,637	578,279	582,288	612,548
*F. nucleatum*	560,132	564,347	570,906	575,682	581,220	619,010	543,114	547,686	557,899	559,287	569,501	578,237
*E.faecalis*	545,878	564,308	576,484	573,064	581,512	603,714	557,411	560,442	562,146	569,102	570,806	594,704
*E. corrodens*	552,590	568,619	579,075	578,317	587,834	608,508	567,342	567,979	578,663	578,561	589,245	589,577
*C. rectus*	532,558	561,741	575,519	577,864	593,144	615,889	544,577	565,388	572,980	569,702	577,294	588,274
*C. gengivalis*	537,625	545,711	556,653	559,280	571,189	583,272	521,654	542,848	556,176	553,238	566,565	578,945
*B. fragilis*	514,676	553,184	567,165	562,438	573,858	600,250	558,006	567,206	572,172	575,094	580,060	597,996
*Aaa*	520,565	531,841	549,008	548,850	565,558	580,608	534,334	535,150	540,761	547,236	552,847	573,088
*C. coli*	528,294	553,992	561,322	558,542	572,180	575,593	553,865	565,098	575,156	571,711	581,769	582,669
*L. casei*	531,608	573,140	583,355	580,824	595,654	604,658	551,711	557,724	57,660	576,619	596,555	599,446

**Table 3:** CMX PU. Minimum (Min), 1st Quartile, median, mean, 3rd Quartile and maximum (Max) of the 40 target species detected in the screw threads and inside the implant.

Thermomechanical Loading							Thermocycling					
	Min	1st Quartile	Median	Mean	3rd Quartile	Max	Min	1st Quartile	Median	Mean	3rd Quartile	Max
*Candida tropicalis*	521,879	526,899	532,735	535,348	540,132	568,524	532,127	544,172	550,925	546,923	553,676	553,717
*Candida glabrata*	509,069	519,093	521,232	524,599	531,062	547,197	523,553	523,558	527,277	528,696	532,414	536,675
*Candida dubliniensis*	502,067	511,557	515,934	516,141	522,602	525,501	523,273	524,197	524,826	525,243	525,872	528,050
*Candida albicans*	505,054	517,696	520,209	519,744	524,708	529,092	518,324	519,122	521,380	521,729	523,987	525,834
*S. pneumoniae*	508,163	509,542	517,133	518,351	524,156	535,767	505,438	512,514	516,580	517,004	521,070	529,418
*S. gallolyticus*	508,370	511,941	512,435	514,700	515,767	524,863	512,751	517,757	520,574	519,627	522,444	524,608
*V. parvula*	498,994	505,425	510,424	509,845	514,491	519,270	509,489	511,837	512,774	515,817	516,755	528,232
*T. denticola*	492,806	510,774	518,132	517,028	525,095	540,757	509,681	510,765	526,990	524,016	530,242	532,403
*T. forsythia*	505,347	519,426	522,475	524,148	529,123	547,926	510,002	515,201	521,239	525,246	531,285	548,505
*S. sobrinus*	595,786	510,908	515,635	520,340	535,644	541,346	516,657	519,578	522,600	526,706	529,727	544,967
*S. sanguinis*	499,460	506,609	509,465	511,159	514,431	525,458	511,951	514,355	515,938	525,292	526,876	557,342
*S. salivarius*	499,330	507,500	512,509	511,075	515,363	519,720	506,010	507,446	500,883	508,136	509,200	509,516
*S. pasteuri*	502,444	507,500	512,509	511,075	515,363	519,720	506,276	513,591	516,103	519,186	521,698	538,260
*S. parasanguinis*	506,311	506,901	507,235	510,155	510,431	523,784	509,446	512,415	516,258	521,968	525,811	545,913
*S. oralis*	502,672	503,168	507,264	513,903	516,239	556,053	509,773	512,126	519,338	519,623	526,834	530,043
*S. mutans*	502,274	506,689	509,707	509,551	510,936	518,002	510,262	511,036	513,776	523,327	526,067	555,495
*S. moorei*	502,459	506,118	508,818	509,641	512,814	521,343	495,690	504,196	512,701	514,469	523,858	535,015
*S. mitis*	496,140	508,397	512,622	510,891	516,285	521,740	509,202	512,173	515,134	515,491	518,452	522,494
*S. constellatus*	499,229	504,608	513,249	510,944	515,374	520,901	502,799	507,690	510,825	517,339	520,474	544,905
*S. aureus*	502,340	506,297	513,276	512,750	516,756	524,366	503,241	510,870	515,086	518,742	522,958	541,558
*P. putida*	502,727	509,252	512,796	516,473	520,938	540,759	513,027	515,250	519,531	525,870	530,151	551,392
*P. nigrescens*	499,713	505,889	514,311	513,822	521,081	526,972	508,985	509,206	512,414	515,173	518,382	526,878
*P. micra*	499,866	509,486	514,798	513,336	518,662	519,904	495,886	505,933	514,731	514,545	523,343	532,833
*P. melaninogenica*	516,074	519,036	521,372	520,954	522,910	526,552	515,292	524,258	528,527	527,933	532,202	539,287
*P. intermedia*	492,805	510,600	512,890	512,164	518,208	520,554	502,698	512,800	516,485	522,552	525,937	553,340
*P. gingivalis*	496,318	510,253	513,687	514,056	519,108	525,049	506,885	508,754	511,323	521,321	523,890	555,753
*P. endodontalis*	500,108	507,253	510,450	510,585	513,594	522,991	510,414	512,129	514,838	518,472	521,181	533,797
*P. anareobius*	499,789	508,881	513,569	512,332	518,974	521,314	510,178	512,684	513,693	521,200	522,209	547,236
*P. aeruginosa*	502,333	504,190	506,289	508,465	512,226	517,909	499,685	504,556	515,162	514,296	524,903	527,176
*M. salivarium*	502,480	503,394	508,459	508,819	513,308	517,638	505,885	509,168	512,009	513,433	516,273	523,829
*K. pneumoniae*	503,118	507,107	509,697	510,426	514,686	517,074	512,896	515,614	516,578	517,487	518,450	523,895
*F. nucleatum*	496,246	509,891	513,442	512,987	517,171	529,364	506,459	506,717	511,210	512,268	516,762	520,195
*E.faecalis*	502,648	509,671	516,600	513,598	517,098	523,633	503,046	508,314	511,641	510,392	513,718	515,240
*E. corrodens*	499,102	507,130	511,786	510,290	514,304	517,051	502,761	507,881	511,606	513,627	517,352	528,535
*C. rectus*	503,240	504,524	508,209	509,771	513,324	521,964	503,162	506,076	508,482	511,234	513,640	524,809
*C. gengivalis*	496,277	509,774	508,597	511,429	518,305	525,247	500,159	502,528	510,465	512,300	520,237	528,113
*B. fragilis*	500,472	506,942	508,975	508,685	511,356	515,116	507,329	509,377	514,210	515,172	520,005	524,938
*Aaa*	500,329	504,265	510,794	510,960	513,583	530,871	503,189	506,050	510,544	512,296	516,790	524,905
*C. coli*	499,754	506,396	513,786	511,665	516,312	521,797	510,199	510,906	513,822	515,704	518,620	528,620
*L. casei*	496,075	507,173	509,809	510,831	515,268	523,264	502,363	508,030	511,699	513,208	516,876	527,070

**Table 4:** CMX U. Minimum (Min), 1st Quartile, median, mean, 3rd Quartile and maximum (Max) of the 40 target species detected in the screw threads and inside the implant.

Thermomechanical Loading							Thermocycling					
	Min	1st Quartile	Median	Mean	3rd Quartile	Max	Min	1st Quartile	Median	Mean	3rd Quartile	Max
*Candida tropicalis*	217,282	341,234	385,222	378,850	439,730	465,562	219,254	254,462	352,510	348,994	447,041	471,700
*Candida glabrata*	225,313	246,065	262,678	268,364	290,845	327,597	184,674	227,776	250,716	242,263	265,203	282,946
*Candida dubliniensis*	285,913	327,206	331,453	348,002	345,835	478,154	327,498	385,702	411,369	434,964	460,630	589,619
*Candida albicans*	110,375	224,104	288,444	272,661	316,789	380,726	301,789	358,219	384,111	378,853	404,745	445,398
*S. pneumoniae*	109,486	143,622	259,004	233,199	287,371	383,126	100,000	212,150	365,828	359,290	512,968	605,506
*S. gallolyticus*	217,038	233,505	290,082	313,556	333,302	586,911	141,288	193,417	258,145	260,751	325,479	385,428
*V. parvula*	143,606	278,304	338,548	309,169	369,346	383,873	124,813	209,168	261,543	248,175	300,550	344,799
*T. denticola*	109,853	224,276	271,285	260,355	327,284	362,279	192,606	202,036	221,284	223,541	242,789	258,989
*T. forsythia*	116,516	228,361	263,827	262,679	296,038	402,735	193,887	202,409	235,870	239,400	272,860	291,973
*S. sobrinus*	110,479	228,074	277,931	275,089	336,756	397,246	262,909	288,476	299,820	301,256	312,601	342,476
*S. sanguinis*	186,712	260,753	319,200	311,340	372,454	413,536	158,952	265,727	369,944	424,695	439,865	439,895
*S. salivarius*	183,190	261,160	289,836	281,151	298,268	368,143	220,188	250,046	279,214	274,872	304,040	320,874
*S. pasteuri*	252,142	287,569	293,603	301,347	300,692	396,385	144,374	170,950	199,114	230,808	258,973	380,631
*S. parasanguinis*	112,858	210,692	301,458	281,694	341,119	399,906	243,447	275,141	303,708	295,559	324,126	331,372
*S. oralis*	280,561	351,924	391,391	390,849	409,087	528,500	297,603	305,743	328,975	360,780	384,012	487,569
*S. mutans*	227,850	290,089	313,220	311,207	376,317	407,205	188,905	277,723	322,566	301,035	345,877	370,103
*S. moorei*	109,937	189,142	243,573	228,104	268,104	306,521	223,630	258,245	289,429	283,920	315,104	333,192
*S. mitis*	150,640	185,939	215,018	243,150	313,548	376,590	154,386	206,231	262,503	252,076	308,348	328,913
*S. constellatus*	145,954	188,560	226,072	250,585	328,127	365,342	193,170	216,380	225,202	227,024	235,847	264,521
*S. aureus*	187,880	262,263	280,057	291,265	302,379	408,494	226,065	308,542	370,618	363,374	425,451	486,196
*P. putida*	143,161	253,023	322,618	309,755	368,265	455,233	297,895	306,702	363,186	366,827	423,311	443,041
*P. nigrescens*	177,730	207,023	266,496	277,380	353,889	397,399	321,529	362,934	386,017	442,321	465,404	675,723
*P. micra*	189,217	190,844	207,689	224,872	243,638	334,089	185,569	265,603	299,005	277,407	310,809	326,048
*P. melaninogenica*	448,694	514,857	561,123	591,295	652,611	880,154	566,416	582,489	605,760	632,014	655,285	750,119
*P. intermedia*	156,879	225,608	287,838	340,493	383,084	812,934	207,091	244,818	320,549	314,632	390,363	410,339
*P. gingivalis*	117,124	264,842	272,863	298,062	369,196	496,776	263,035	268,326	281,348	302,660	315,681	384,908
*P. endodontalis*	110,453	233,541	284,995	268,700	331,040	345,795	147,782	176,117	204,091	242,392	270,366	413,606
*P. anareobius*	151,960	225,042	265,177	263,778	293,364	405,937	146,384	230,859	280,924	279,409	329,475	409,404
*P. aeruginosa*	227,437	239,907	290,105	308,114	377,696	413,180	225,416	225,727	246,442	273,559	294,274	375,936
*M. salivarium*	187,273	247,973	341,173	333,144	415,076	469,885	309,088	333,366	364,032	365,248	395,914	423,843
*K. pneumoniae*	195,112	282,004	404,784	402,078	450,658	783,747	232,059	260,969	348,822	358,690	446,544	505,059
*F. nucleatum*	150,975	272,399	305,683	303,963	341,448	454,396	235,202	263,872	306,925	317,749	368,002	421,946
*E.faecalis*	186,020	274,720	341,316	325,884	396,734	420,810	309,552	331,993	360,781	353,452	382,240	382,694
*E. corrodens*	231,374	281,719	336,372	366,000	409,572	574,781	230,021	282,075	343,949	333,581	395,455	416,406
*C. rectus*	328,256	351,452	426,916	477,979	581,944	767,913	115,174	209,127	361,902	343,182	495,957	533,751
*C. gengivalis*	233,038	310,492	342,198	425,489	541,128	748,697	236,263	260,985	287,295	320,936	347,246	472,891
*B. fragilis*	277,918	292,194	355,0285	408,566	432,525	792,708	348,069	370,687	402,494	470,874	502,680	730,439
*Aaa*	110,516	268,966	287,284	296,592	338,523	427,716	148,299	178,126	229,103	244,087	295,064	369,845
*C. coli*	147,005	230,240	268,391	279,505	346,504	389,418	268,353	293,782	306,211	329,242	335,372	410,996
*L. casei*	147,514	282,125	345,747	319,614	377,600	387,103	147,246	233,982	322,463	322,463	346,930	595,550

## Data Availability

The data that support the findings of this study are available from the corresponding authors upon reasonable request.
